# Conserving goat sperm post-thawed gene expression and cellular characteristics using the antioxidant coenzyme Q10 supplementation

**DOI:** 10.14202/vetworld.2024.1637-1647

**Published:** 2024-07-30

**Authors:** Yudit Oktanella, Imam Mustofa, Fahrunnisak Al-Firda Razak An-Haru, Desinta Dwi Melati Putri, Viski Fitri Hendrawan, Suherni Susilowati, Nurhusien Yimer Degu, Tatik Hernawati

**Affiliations:** 1 Department of Veterinary Reproduction, Faculty of Veterinary Medicine, Brawijaya University, Malang, East Java, Indonesia; 2 Department of Veterinary Reproduction, Faculty of Veterinary Medicine, Airlangga University, Jl. Dr. Ir. H. Soekarno, Mulyorejo, Kec. Mulyorejo, Surabaya, East Java, Indonesia; 3 Veterinary Clinical Studies, Faculty of Veterinary Medicine, University Putra Malaysia, Serdang, Selangor Darul Ehsan, Malaysia; 4 Faculty of Veterinary Medicine, Airlangga University, Jl. Dr. Ir. H. Soekarno, Mulyorejo, Kec. Mulyorejo, Surabaya, East Java, Indonesia

**Keywords:** antioxidant, gene expression, goat sperm, semen diluent

## Abstract

**Background and Aim:**

The use of frozen goat semen for artificial insemination frequently results in a decline in sperm quality following thawing, which can be attributed to cold shock from cryopreservation, reduced motility, and possible DNA damage. Freezing may compromise mRNA stability due to the presence of free radicals. Despite strong post-thaw motility and no visible DNA fragmentation, sperm can still exhibit altered gene expression patterns. To reduce the damaging impact of free radicals during cryopreservation, antioxidants are typically added to the freezing medium. This study assessed the impact of adding coenzyme Q10 (CoQ10) to frozen sperm diluent on the ATP5F1A and CPT2 gene expression, sperm motility, and viability post-thawing.

**Materials and Methods:**

CoQ10 was added to sperm at six different concentrations: 0 mg/dL (P0), 6.25 mg/dL (P1), 12.5 mg/dL (P2), 25 mg/dL (P3), 50 mg/dL (P4), and 100 mg/dL (P5). The Statistical Package for the Social Sciences (SPSS) software version 22 was used to conduct comparative tests using one-way analysis of variance followed by Duncan’s test for motility and viability and Kruskal–Wallis test followed by pairwise comparison test for membrane integrity and gene expression.

**Results:**

The addition of CoQ10 to semen diluent has a notable impact on the post-thawed quality of sperm. The most significant outcomes were observed with a 25 mg/dL dosage (P3) for cell viability, membrane integrity, and ATP5F1A gene expression, and with a 50 mg/dL dosage (P4) for sperm motility, membrane integrity, and CPT2 gene expression.

**Conclusion:**

Incorporating CoQ10 into frozen semen diluent improves gene expression and prevents deterioration of the cell quality of thawed goat spermatozoa. While the study demonstrates the benefits of CoQ10, the precise molecular mechanisms through which CoQ10 enhances gene expression and cell quality were not fully elucidated. Further investigation is needed to understand these mechanisms in detail. Comparative studies with other antioxidants and cryoprotectants can help establish the relative efficacy of CoQ10 and potentially develop more effective combinations.

## Introduction

In bucks, frozen semen used for artificial insemination often results in decreased sperm quality post-thawing [1]. The primary cause of post-thawing sperm quality decrease is cryopreservation. Thawing can negatively impact sperm quality and viability [2]. 40%–46% of pregnancy rates can be attributed to poor quality frozen goat semen used for reproductive processes and artificial insemination preparations [3]. The freezing process, characterized by holding, dilution, equilibration, and storage, may negatively impact semen quality.

The quality of frozen goat semen is significantly reduced compared to fresh semen. A suitable diluent should prevent cold shock, maintain optimal semen pH, and supply essential sperm nutrients [4]. Ions, lipids, proteins, and carbohydrates in sperm can be influenced by cryopreservation [5].

Disruptions in the sperm antioxidant defense system during cryopreservation, as well as the activation of L-amino acid oxidase in dead or defective cryopreserved sperm, strongly contribute to the increased reactive oxygen species (ROS) production detected in ruminant sperm after freezing-thawing, with the sperm plasma membrane serving as the primary site of ROS-induced damage [6, 7]. Cryopreservation has been shown to decrease the expression of critical pathways and genes in sperm, leading to DNA damage and morphological distortion [8]. Long-term sperm storage in liquid nitrogen led to an increased number of abnormalities [9]. Hezavehei *et al*. [10] discovered that cryopreservation impacts sperm mRNA stability, protein and gene expression, and epigenetics. Cryopreservation significantly suppresses the mRNA expression of multiple proteins as indicated in oyster [11] and bull [12] sperm. Furthermore, the transcriptional abundances of many genes are altered in cryopreserved spermatozoa, leading to changes in pathways essential for sperm function and fertility [13]. The precise mechanisms of how frozen sperm undergo gene expression change and maintain DNA integrity remain unclear [14].

Effective cryopreservation hinges on accurately managing the ATP synthase and CPT2 genes in sperm. ATP synthase proteins create ATP from ADP within the oxidative phosphorylation process. ATP synthase protein comprises the F1 and F0 components. The F1 domain of ATP synthase in the mitochondrial matrix functions as the catalytic component due to its hydrophilic nature [15]. The component consists of nine subunits: three α-subunits, three β-subunits, γ, δ, and ε. The alpha subunit functions as a substrate-binding site for the beta subunit. The gene ATP5F1A is responsible for encoding the F1 alpha subunit of ATP synthase, which is integrated into the inner mitochondrial membrane as part of the ATP synthase complex [16].

In addition to ATP synthesis, CPT2 plays a crucial role in energy production. CPT2 encodes for carnitine palmitoyltransferase, which transports long-chain fatty acids into the mitochondria for β-oxidation and ATP production. This gene is mostly active in tissues with high energy demands, such as the heart, liver, and skeletal muscle. Recent study hint at CPT2 gene expression being crucial in sperm cells [17]. Optimizing cryopreservation requires a comprehensive understanding of the regulation and expression of the ATP synthase and CPT2 genes in sperm.

Novel insights have led to improved sperm cryopreservation methods, including the introduction of proteins, antioxidants, and cryoprotective chemicals into the freezing medium. Although significant strides have been made, they are not sufficient to restore full viability of all sperm following cryopreservation. [10]. Improving sperm cryopreservation outcomes in ruminant animals, including cattle, sheep, goats, buffaloes, deer, and other wild species poses a significant challenge. This group of mammals, which are found all over the world, provides essential food supplies (meat and milk) and helps to maintain sustainable agriculture because of their capacity to feed on fibrous vegetables or byproducts that cannot be used as human food [18].

Antioxidants such as vitamin C [19], quercetin [20], epigallocatechin-3 gallate [21], and vitamin E [22] can be incorporated into sperm extenders. The study proposed that this type of antioxidant significantly impacts the mitochondrial respiratory chain, gene expression, and various cellular processes, including signaling, transport, and metabolism [2, 23]. Antioxidants added during sperm cryopreservation may help preserve normal gene expression patterns by maintaining mRNA integrity. Adding antioxidants to the semen extender purportedly enhances gene expression in buck spermatozoa, which is claimed to lead to increased fertility and reproductive success through supposedly reduced oxidative stress. Antioxidants in semen extenders can help prevent lipid peroxidation, thus preserving sperm cell membranes and maintaining their health. This antioxidant-based semen extender modification could revolutionize goat sperm storage and artificial insemination.

This study aimed to investigate the impact of adding coenzyme Q10 (CoQ10) to frozen sperm diluent on the expression of the genes ATP5F1A and CPT2, as well as the motility, viability, and membrane integrity of spermatozoa upon thawing.

## Materials and Methods

### Ethical approval

This study was conducted in accordance with the Research Ethics Committee of Brawijaya University, Malang, Indonesia. The approval was obtained from the Chair of the Brawijaya University Research Ethics Commission, as stated in the Statement of Ethical Clearance Number 055-KEP-UB-2023.

### Study period and location

The study was conducted from July to September 2023. Data collecting techniques for fresh semen up to viability and motility were conducted at Teaching Farm Fakultas Kedokteran Hewan, Universitas Airlangga, Kecamatan Kedamean, Kabupaten Gresik, Jawa Timur, while data collection procedures for ATP5F1A and CPT2 gene expression up to data processing were conducted at Animal Disease Diagnose Laboratory Fakultas Kedokteran Hewan, Universitas Brawijaya, Kecamatan Dau, Kabupaten Malang, Jawa Timur.

### Semen extender preparation

Frozen semen production was initiated by making a diluent of semen extenders A and B. 100 mL distilled water, 10% skim milk, 5% egg yolk, 0.1% penicillin (1000 IU), 0.75% fructose, and 0.1 g/100 mL streptomycin were combined to make Semen extender A. 100 mL of distilled water was added to skim milk and heated indirectly at 92°C–95°C for 10 min and then placed in a water jacket to cool it down to 37°C. The magnetic stirrer was used to homogenize the separated egg yolk after filtration. Antibiotics and fructose were directly added to the homogenized diluent mixture. The subsequent action entails diluting B with 2% glucose, 12% glycerin, and varying CoQ10 concentrations solution: 0, 6.25, 12.5, 25, 50, and 100 mg/dL. Manually mixed glucose and glycerin in each stage and then placed the mixture on a magnetic stirrer. Subsequently, CoQ10 was introduced into diluents with different ratio concentrations, 0 mg/dL (P0), 6.25 mg/dL (P1), 12.5 mg/dL (P2), 25 mg/dL (P3), 50 mg/dL (P4), and 100 mg/dL (P5). The specified ratio of the diluents was achieved by adding sterile distilled water.

### Fresh semen collection

Fresh sperm originated from a 3-year-old excellent buck from the Kedamean Village Livestock Group in Gresik Regency, East Java, Indonesia, was used for the study. Fresh semen was collected twice a week using an artificial vagina, and only the third or fourth ejaculations with high concentration were frozen.

### Frozen semen preparation

The process of creating frozen semen starts by evaluating the quality of fresh semen, which involves an examination of volume, texture, color, odor, pH levels, concentration, viscosity, and the percentage of viable/non-viable sperm. After the assessment, semen was diluted by adding extender A at room temperature (37°C). In glycerolization, the semen-extender combination was cooled down to 3°C–5°C over an hour. A suitability assessment was conducted on the semen before freezing. This includes assessing motility, viability, abnormalities, and membrane integrity of spermatozoa. Once completed, the straws were printed, sealed, and placed on a rack. The canister containing the straws was cooled from 4°C to –160°C for 11 min before freezing. During freezing storage, the liquid nitrogen temperature remained at –196°C.

### Post-thawing examination

#### Sperm membrane integrity

The hypoosmotic swelling test was used to evaluate the membrane integrity of goat spermatozoa post-thawing [24]. To balance the external fluid conditions, the spermatozoa were immersed in a hypoosmotic solution that increased their internal water volume. A hypoosmotic solution was prepared by adding 0.735 g sodium citrate dehydrate and 1.351 g D-fructose to 100 mL of distilled water. The HOST test procedure started by thawing frozen semen with water at 37°C for 10 s. Next, 1 mL of hypoosmotic solution was put into the object glass and added with 0.1 mL of spermatozoa continued with incubation at 37°C for 30 min. Subsequently, samples were observed under a light microscope with 400× magnification to assess a typical change, specifically, swelling of the tail at the tip [24].

#### Sperm’s motility

After thawing, the motility of goat sperm was examined in six samples treated with different treatments using a computer-assisted sperm analysis (CASA) device equipped with SpermVision™ software (Minitube Hauptstraβe 41, 8414 Tiefenbach, Germany). The examination process involved thawing the semen in water at 37°C for 10 s, followed by dropping it on a glass slide and adding an extender at a ratio of 1:29. Subsequently, observation was conducted using a microscope with magnifications of 400× and 1000× connected to CASA, using settings outlined in Table-1 [25] to calculate the total number of viable and progressively moving spermatozoa.

**Table-1:** CASA settings for goat spermatozoa analysis [25].

Parameters	Settings
Frame capture rate (Hz)	60
Frame count	30
Head size maximum (µm^2^)	70
Head size minimum (µm^2^)	6
Maximum elongation (%)	100
Elongation minimum (%)	1
Slow VAP (µm/s)	20
Slow VSL (µm/s)	30
Maximum photometer	70
Minimum photometer	60
Progressive STR	80
Progressive VAP (µm/s)	30
Static VAP (µm/s)	4
Static VSL (µm/s)	1
Head brightness minimum	200
Tail brightness minimum	96
Maximum width-to-length ratio (%)	90
Minimum width-to-length ratio (%)	1
Temperature (°C)	37

CASA=Computer-assisted sperm analysis; VAP=Average path velocity: VSL=Straight line velocity; STR=Straightness

#### Sperm’s viability

Viability examination of post-thawing goat spermatozoa was conducted on six samples with different treatments using a combination of eosin-nigrosin staining method [9] and microscope reading with the assistance of CASA (CASA®; WI-LI New Century Technical Development, Tiefenbach, Germany) device. The examination began by melting the semen in water at 37°C for 10 s. Subsequently, an eosin-nigrosin solution was added to the melted semen in a 1:1 ratio and then slowly homogenized. The sample was then dropped onto the glass object, and a smear was applied by pushing or pulling until a thin layer was formed on the surface. The sample smear was dried at room temperature (27°C–29°C). Spermatozoa viability was observed using a microscope at 400× and 1000× magnification levels. Viability calculations were performed with the assistance of CASA software. Spermatozoa heads stained with eosin-nigrosin solution were considered dead, whereas those unstained were considered alive [9].

### RNA extraction

RNA extraction was performed using the RNA extraction kits (RNA extraction kits direct-zol™ product: Zymo Research®, California, USA). The extraction process involved several steps, including adding DNA/RNA Shield^™^ (RNA extraction kits direct-zol™ product: Zymo Research®). to the thawed semen, followed by centrifugation and washing with RNA Lysis Buffer^™^ (RNA extraction kits direct-zol™ product: Zymo Research®). and absolute ethanol. After digestion with DNase 1 and DNA digestion buffer, the samples were washed with RNA wash buffer before elution in DNase/RNase-Free Water. Finally, the extracted solution was stored for further analysis using a spectrophotometer Nanodrop®ND-1000 (Thermo Fisher Scientific: Massachusetts, USA) to determine the DNA content.

### cDNA synthesis

cDNA synthesis from RNA samples was performed using the cDNA synthesis kits (iScript^™^ cDNA Synthesis Kit: Bio-Rad, California, USA). The process began by thawing the RNA extraction results at temperatures below 4°C. Microtubes were filled with 2 μL of 5× g DNA Buffer™ (iScript^™^ cDNA Synthesis Kit: Bio-Rad), 3 μL of extracted RNA, and 5 μL of Rnase-Free ddH_2_O™ (iScript^™^ cDNA Synthesis Kit) before homogenization. The solution was then incubated at 42°C for 3 min. Meanwhile, a reverse transcription reaction component was prepared by combining specific reagents for each treatment sample. The resulting mixture was added to the incubated solution and mixed slowly. Subsequently, the solution was subjected to cDNA amplification at specified temperatures in a T100 Thermal Cycler™ (Bio-Rad) for set durations. The post-amplification cDNA concentration was determined using a spectrophotometer Nanodrop®ND-1000 (Thermo Fisher Scientific) to guide subsequent gene expression analysis.

### Reverse transcription polymerase chain reaction (RT-PCR)

Gene expression was analyzed using RT-PCR with a CFX96^™^ Touch Real-Time PCR detection system (Bio-Rad) with specific primers, as detailed in Table-2. The process began by mixing each primer solution with Tris-EDTA (TE) Buffer, followed by a 3-min incubation and homogenization using a vortex for 1 min. A certain amount of each primer was then diluted to 10 pmol from the original 100 pmol concentration. Subsequently, 0.5 μL of each forward and reverse primer were combined in a new microtube with 5 μL of Evagreen^™^ (Humanizing Genomics Macrogen®, Seoul, South Korea) and 1 μL of ddH2O. After homogenization using vortex and spin-down methods, the material was transferred to a PCR tube containing the cDNA sample (3 μL). The resulting mixture was then inserted into a Bio-Rad CFX96 RT-PCR system for analysis. Reading of gene expression for 1 h, including pre-denaturation at 95°C for 3 min, denaturation at 95°C for 10 s, annealing for 30 s, extension at 72°C for 30 s, final extension at 65°C for 5 s, post-extension at 95°C for 5 min, and 35 cycles. Hydroxymethylbilane synthase is a housekeeping gene that can be used as an endogenous control indicator to determine normal gene expression.

**Table-2:** Primer sequence information.

Gene	GenBank accession number	Primer sequence (5’- 3’)	Annealing temperature (°C)	Amplicon size (bp)
ATP5F1A	XM_018039617.1	F: TCTGAACTTGGAGCCTGAC	49.4	108
		R: ACTGGAACATCTACAATAGCCC		
CPT2	XM_018044297.1	F: AGTCAGTAGGCACTATTCTGTG	49.1	165
		R: CCTGAAAATGTCCTTAAAACCG		
Hydroxymethylbilane synthase	XM_005689536.3	F: CTTGCCAGAGAAGAGTGTGG	50	116
		R: CAGCCGTGTGTTGAGGTTTC		

### Statistical analysis

Statistical analysis was conducted using IBM Statistical Package for the Social Sciences 25.0.10 for Windows (IBM Corp., NY, USA). All examination data were subjected to normality and homogeneity tests using the Shapiro–Wilk and Levene tests. Spermatozoa function variables, including motility and viability, were analyzed using a one-way analysis of variance (ANOVA) followed by the Duncan test. At the same time, membrane integrity, ATP5F1A, and CPT2 gene expression was examined using the Kruskal-Wallis test, followed by the Pairwise test. The correlation between ATP5F1A/CPT2 gene expression and sperm function was assessed using the Spearman Rank test at a 95% confidence level (ɑ = 0.05).

## Results

### Fresh semen evaluation

An examination of fresh semen and the characteristics of goat spermatozoa was conducted to assess the sample’s suitability for cryopreservation or freezing as well as to calculate the appropriate extender. Based on the feasibility study results (Table-3), the semen collected using the artificial vagina method was suitable for cryopreservation due to its good quality and meeting all criteria.

**Table-3:** Fresh semen evaluation.

Parameter	Result	Reference
Volume (mL)	0.9 mL	0.1–1.5 mL [26]
Odor	Specific	Specific [24]
Viscosity	Very viscous	Viscous [24]
pH	6–7	6.4–7.2 [26]
Color	White	Creamy white [27]
Concentration	2.293×10^6^/mL	2.500–3.000× 10^6^/mL [28]
Mass motility	++	++/good - +++/very good [27]
Individual motility	85%	≥ 70% [26]
Viability	90%	≥ 70% [26]

### Post-thawing spermatozoa viability

**Figure-1:**
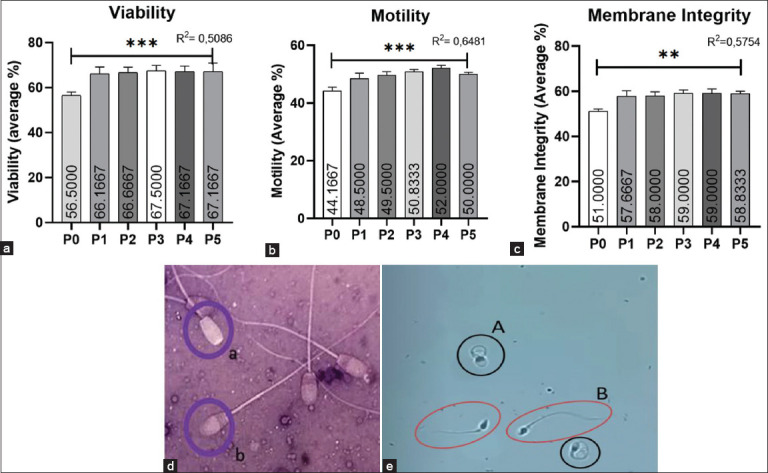
Microscopic examination of post-thawed frozen semen. (a) Average post-thawing spermatozoa viability for each treatment group, as determined using the analysis of variance (ANOVA) test which shows (***) = p < 0.0001. (b) Average post-thawing spermatozoa motility for each treatment group, as determined using the ANOVA test which shows (***) = p < 0.0001. (c) Average post-thawing spermatozoa membrane integrity for each treatment group using the Kruskal–Wallis test, which indicated (**) = p < 0.03. (d) Image of (a) live and (b) dead sperm on eosin-nigrosin staining. (e) Image of (a) a swollen tail and (b) no swollen tail by HOS test. P0 (CoQ10 0 mg/dL), P1 (CoQ10 6.25 mg/dL), P2 (CoQ10 12.5 mg/dL), P3 (CoQ10 25 mg/dL), P4 (CoQ10 50 mg /dL), and P5 (CoQ10 100 mg/dL). ANOVA=Analysis of variance.

The analysis of post-thawing viability examination data revealed a significant effect (p < 0.05) in the CoQ10+ group compared with the negative control group or when using semen extender without adding CoQ10, as shown in Figure-1. Upon observing live and dead spermatozoa, it is evident that there is minimal difference between treatment groups 1 and 5 in terms of average viability. P3 (CoQ10 25 mg/dL) exhibited the highest average viability at 67.5%.

### Post-thawing spermatozoa motility

The results of examining post-thawing spermatozoa motility using CASA are presented in Table-4 and Figure-1. ANOVA indicated a significant effect (p < 0.05) on the examination of post-thawing spermatozoa motility. According to Duncan’s test, it was found that the negative control group had the lowest average progressive motility at 44.2% ± 1.33%, which was significantly different (p < 0.05) from all other groups (P1–P5). In addition, group P2 showed significant differences (p < 0.05) compared with the negative control and P4 groups but not compared with P1, P3, and P5 (p < 0.05). Meanwhile, group P4 exhibited a progressive motility average of 52% ± 1.09%, significantly different from all treatment groups except for group P3. The highest average motility of the sperm was observed in P4 with CoQ10 at a concentration of 50 mg/dL, with an average of 52% ± 1.09%.

**Table-4:** Post-thawing goat sperm examination.

Treatment groups	Gene expression of ATP5F1A ± SD**	Gene expression of CPT2 ± SD**	Average motility (%) ± SD*	Average viability (%) ± SD*	Average membrane integrity (%) ± SD**
P0 (CoQ10 0 mg/dL)	32.90 ± 1.46^a^	36.55 ± 2.90^a^	44.2 ± 1.33^a^	56.5 ± 1.52^a^	51.00 ± 1.09^a^
P1 (CoQ10 6.25 mg/dL)	32.35 ± 1.76^a^	36.52 ± 0.67^a^	48.5 ± 1.87^b^	66.2 ± 2.92^b^	57.67 ± 2.58^b^
P2 (CoQ10 12.5 mg/dL)	33.24 ± 0.37^b^	37.29 ± 1.37^a^	49.5 ± 1.38^bc^	66.7 ± 2.34^b^	58.00 ± 1.79^b^
P3 (CoQ10 25 mg/dL)	33.41 ± 0.52^b^	40.21 ± 0.72^b^	50.8 ± 0.75^cd^	67.5 ± 2.34^b^	59.00 ± 1.55^b^
P4 (CoQ10 50 mg/dL)	32.90 ± 0.32^a^	40.40 ± 0.64^b^	52.0 ± 1.09^d^	67.2 ± 2.32^b^	59.00 ± 2.00^b^
P5 (CoQ10 100 mg/dL)	33.14 ± 0.02^a^	39.71 ± 0.85^b^	50.0 ± 0.63^bc^	67.2 ± 3.76^b^	58.83 ± 1.17^b^

(*)=statistical analysis using one-way analysis of variance followed by Duncan’s test; (**)=Statistical analysis using Kruskal–Wallis test followed by Pairwise comparison. Superscripts ^a,b,c,d^indicate significant differences (p < 0.05) in each parameter (column), SD=Standard deviation

### Post-thawing sperm membrane integrity

Examination of the membrane integrity of post-thawing goat spermatozoa showed a significant effect (p < 0.05) in the group with CoQ10 compared with the group without it (Table-4 and Figure-1). The average value of membrane integrity was 51.13% for the negative control group. The P3 and P4 groups had the highest average membrane integrity value of 59%, which was significantly different from the control group but not the P1, P2, and P5.

### Gene expression

The expression of ATP5F1A and CPT2 was examined using RT-quantitative PCR to yield quantitative data. Data obtained from the quantification cycle (Cq value) in amplification curve were then subjected to statistical analysis. The results of the Kruskal–Wallis test revealed a significant difference (p < 0.05) in examining the expression of the ATP5F1A and CPT2 genes. The pairwise comparison revealed differences in the expression of ATP5F1A among the various treatment groups. The highest expression was observed in P3 (CoQ10 25 mg/dL). Similarly, significant differences were found between several treatment groups for CPT2, with the highest expression observed in P4 (CoQ10 50 mg/dL).

## Discussion

Cryopreservation is a popular method for storing semen for artificial insemination. Drastic temperature changes, ice crystal formation, and stress conditions can negatively impact spermatozoa. These effects can negatively impact motility, viability, and membrane integrity. Cryopreservation also influences the transcriptomic composition of RNA products [36]. The researcher suspects that the addition of antioxidants into semen diluent can reduce oxidative stress caused by excessive ROS production. This study assessed the influence of CoQ10 on sperm quality features, including motility, viability, membrane integrity, transcription products, and gene expression of ATP5F1A and CPT2.

In this study, the treatment group (P4, CoQ10 50 mg/dL) exhibited a significantly greater average progressive motility (52%) than the negative control group. CoQ10 improved sperm motility in various species including cows, buffaloes, sheep, chickens, and groupers [29–33]. Gardela *et al*. [34] documented the detrimental effects of CoQ10 at 100 μM and 200 μM concentrations with rabbit semen stored at sub-zero temperatures. Excessive antioxidant consumption can lead to the development of hazardous redox forms that harm spermatozoa [35]. CoQ10 acts as a cofactor and ion transporter from protein chain complex I (nicotinamide adenine dinucleotide hydrogen [NADH]: Ubiquinone reductase) and complex II (succinate: Ubiquinone reductase) to complex III (ubiquinol: Cytochrome c reductase) during the electron transport stage of ATP formation in the mitochondrial membrane [36]. CoQ10 can aid in reducing mitochondrial membrane damage by minimizing ROS buildup in ATP generation and regenerating alpha-tocopherol radicals into antioxidant alpha-tocopherol [37].

Motility examination revealed P5 had lower CoQ10 levels (100 mg/dL) compared to P4 (50 mg/dL). A study by Gardela *et al*. [34] revealed that adding 100 μM and 200 μM of CoQ10 to a semen extender with frozen rabbit semen had a detrimental effect. 1.5 μM CoQ10 decreased both total and progressive motility than 1 μM in the study by Yousefian *et al*. [38]. Decreases can occur due to the concentration of CoQ10, which exceeds the threshold of cell needs for antioxidants; thus, CoQ10 can turn into toxic compounds [32] In carrying out its role in the mitochondrial inner membrane, CoQ10 is completely reduced to ubiquinol (CoQ10H2) and oxidized back to ubiquinone (CoQ10) to allow the ion transport process to proceed [36]. If the amount of CoQ10 is excessive, CoQ10 will still be reduced but only partially to ubisemiquinone (CoQ10H), a toxic redox form that causes negative effects on spermatozoa [32, 35].

Sperm viability plays a crucial role in sperm-oocyte activation, fertilization, and embryonic development [39]. This viability evaluation revealed that the addition of CoQ10 substantially affected all treatment groups compared with the negative control group. P3 (CoQ10 25 mg/dL) had the most excellent mean viability (67.5%). Wafa *et al*. [29] found that adding 0.03 μM CoQ10 to frozen buffalo semen extender improved viability to 65.5%. 25 μM CoQ10 decreases NO2, H2O2 concentrations, and viability, as reported by De Albuquerque Lagares *et al*. [40]. Research suggests that CoQ10 positively affects bull semen [29, 30] and chicken reproductive systems [32, 37].

Preserving membrane integrity is essential for sperm motility, viability, and transport function [41]. Healthy membrane integrity is critical for managing spermatozoa’s motility, viability, and transport system [41]. Membrane integrity influences sperm capacitation, acrosome reaction, and sperm-oocyte fusion [42]. CoQ10 added to all treatment groups significantly improved membrane integrity compared to the negative control group. P3 and P4 groups had the highest average membrane integrity of sperm (59%), while CoQ10 supplementation could potentially preserve this value [41]. In various animal semen investigations, the addition of CoQ10 was able to preserve the quality of post-thawing spermatozoa membrane integrity, such as in horses (73.1%) [40], buffalo (63.3%) [30], and cattle (63.1%) [30]. Individual differences in cattle, extender concentration, and thawing technique can all alter percentages of spermatozoa membrane integrity [43].

CoQ10 is an antioxidant that can preserve spermatozoa cell membranes. CoQ10’s lipophilic or fat-soluble nature allows this antioxidant to quickly diffuse directly into the structure of unsaturated or polyunsaturated fatty acids (PUFA) in sperm, nuclear, and organelle membranes. CoQ10’s antioxidant function is enhanced due to the reduction in lipid peroxidation instigated by free radicals [44]. CoQ10 is often located in the hydrophobic portions of the phospholipid bilayer in all biological cell membranes. CoQ10 can prevent cell death directly or function as an antioxidant. Alteration of the isoprenyl structure in CoQ10 leads to changes in membrane protein interactions and subsequently influences membrane stability [35]. As an antioxidant, CoQ10 works in the active form of ubiquinol by inhibiting the initiation and propagation stages of lipid peroxidation in the phospholipid bilayer membrane through a donor mechanism or as a recipient of free electrons from free radicals, resulting in the formation of more stable molecules that do not cause lipid oxidation. This process reduces the quantity of ROS in the form of OH- and -O2 [35]. This antioxidant can regenerate alpha-tocopherol radicals into alpha-tocopherol, thereby serving as an antioxidant that protects cell membranes. CoQ10 also suppresses the opening of mitochondrial transition pores (mPTPs). Opening the mPTP can trigger mitochondrial depolarization, migration of mitochondrial components to the cytosol, and even the release of cytochrome C, which signals the initiation of the apoptotic cell death pathway [36]. CoQ10 responsibilities preserve membrane integrity and viability in post-thawing spermatozoa while suppressing quality loss.

A linear relationship exists between viability and membrane integrity. Membrane integrity also affected viability in this study. According to Bahmid *et al*. [44], plasma membrane integrity is necessary to ensure survival in spermatozoa. The findings demonstrated that P1 (CoQ10 6.25 mg/dL) had the most favorable outcomes when CoQ10 was added to the assessment of membrane integrity and viability, even though it did not differ significantly from the other treatments. The most likely reason that these results occur is that the function of CoQ10 in maintaining membrane integrity is influenced by other factors, such as enzymes that reduce CoQ10 to be able to actively work in the form of ubiquinol (CoQ10H2) as an antioxidant and maintain the membrane phospholipid bilayer. Several enzymes can play a role in reducing CoQ10 in the plasma membrane, including NADH-cytochrome b5 reductase and NAD (P)H-quinone oxidoreductase 1 (NQO1) [36].

**Figure-2:**
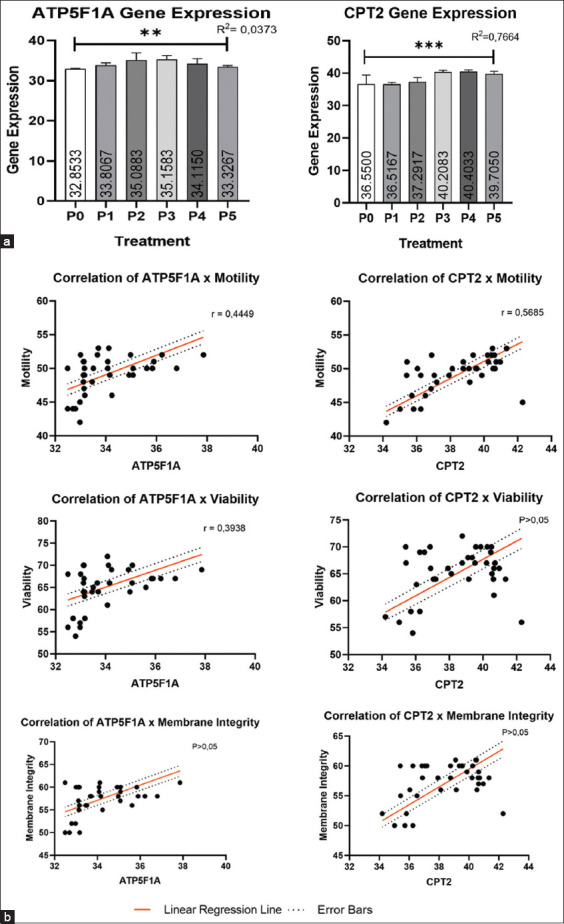
Results of the Kruskal–Wallis test (**) = p < 0.01; (***) = p < 0.005) and correlation analysis between (a) ATP5F1A and (b) CPT2 gene expression and motility, viability, and membrane integrity using the Spearman Rank test.

Examination of mRNA gene expression in sperm can provide insights into the spermatogenesis process, sperm function, quality, and impact on fertilization success and zygote development [45]. The gene ATP5F1A encodes the F1 alpha subunit of mitochondrial ATP synthase, located in the cristae and inner membrane. ATP synthesis in cells relies on this protein [16]. The inner mitochondrial membrane contains the protein CPT2. During fat oxidation, it facilitates the conversion of acylcarnitine to acyl-CoA, enabling energy production through ATP formation through oxidative phosphorylation [46]. The ATP5F1A and CPT2 genes showed significant differences (p < 0.05) in all treatment groups using the Kruskal-Wallis test. The highest expression of the ATP5F1A gene in the pairwise comparison test result was observed in P3 (CoQ10 12, 5 mg/dL), but it was not significantly different from that in P2 (CoQ10 25 mg/dL). For CPT2, the highest expression was observed in P4 (CoQ10 50 mg/dL) but was not significantly different from that in P3 (CoQ10 25 mg/dL) and P5 (CoQ10 100 mg/dL) (Figure-2).

CoQ10’s antioxidant role in sperm cell membranes contributes to the upregulation of these two genes. Adding antioxidants to frozen semen extenders is vital to prevent protein translation issues. CoQ10, an antioxidant, stabilizes and inhibits cell membranes by donating or attracting free electrons from free radicals. This process leads to the formation of new, more stable molecules that reduce excessive lipid oxidation and prevent DNA damage in spermatozoa cells [47]. CoQ10 has the potential to support gene expression by blocking the ceramide molecule, thus inhibiting DNA binding and preventing the activation of transcription factors that control gene regulation. CoQ10 administration can also assist gene expression by inhibiting tyrosine kinase activation by ROS. This modifies gene expression, resulting in the translation of functional proteins from the expressed product [36]. However, it is necessary to carry out a more in-depth study regarding the action of CoQ10 directly on the expression of products in the form of mRNA. Meanwhile, the results obtained in the examination of ATP5F1A gene expression in which the addition of higher doses of CoQ10 from P2 (CoQ10 12.5 mg/dL) and P3 (CoQ10 25 mg/dL) tended to yield smaller results than the two treatments. The change of CoQ10 into toxic ubisemiquinone (CoQ10H) [35] probably explains the reduction in gene expression products and mRNA. A more comprehensive investigation into the influence of CoQ10 on mRNA product expression is required.

During sperm maturation, ATP5F1 transcription supports the function and quality of spermatozoa [45]. This study observed a correlation between ATP5F1A gene expression and sperm quality, including motility, viability, and membrane integrity, to establish the relationship between ATP5F1A gene expression and cellular characteristics in post-thawing goat sperm. The correlation between ATP5F1A gene expression and motility and viability was significant (p < 0.05), but not substantial with membrane integrity (p > 0.05), as shown by the Spearman Rank correlation test. The correlation between ATP5F1A gene expression and motility was relatively weak, with a coefficient of 0.4449. According to Schober *et al*. [48], the ATP5F1A gene’s expression exhibited a weak correlation with a coefficient of 0.3938. The moderate correlation between ATP5F1A gene expression and motility may be attributed to the sole role of the functional protein produced from the translation of the ATP5F1A gene, which acts as an ATP-forming component in the oxidative phosphorylation pathway by combining with other proteins to form ATP synthase [16]. The ATP produced during oxidative phosphorylation is considered the most significant energy source that can affect the motility of sperm [49]. Other factors that can affect spermatozoa motility include the expression of genes that form the ATP synthase protein, such as ATP5B, ATP6, ATP8, and other genes, as well as the structural integrity or abnormalities of spermatozoa [50–52].

The weak correlation between the expression of the ATP5F1A gene and the viability of post-thawing spermatozoa may be due to cells fulfilling their ATP requirements for metabolism not only through oxidative phosphorylation but also through glycolysis, which does not necessitate the production of the ATP synthase protein [49]. Factors including ultrastructural changes, biochemical components, metabolism, and cell function significantly impact sperm viability [53]. The expression of ATP5F1A did not affect membrane integrity. Spermatozoa membrane integrity can be impacted by various factors, including stress conditions (traumatic, chemical, oxidative, and osmotic), temperature changes, osmolarity shifts, environmental pH variations, and ice crystal formation. These factors can impair both the viability and membrane integrity of spermatozoa [5].

The CPT2 gene is the gene encoding the regulatory enzyme CPT2, which has the function of catalyzing the conversion of acyl groups from acylcarnitine to acyl-CoA during the oxidation of fatty acids to form ATP [54]. The study investigated the link between CPT2 gene expression and sperm motility, viability, and membrane integrity through a correlation test. The Spearman rank test revealed that CPT2 gene expression significantly correlated with motility (p < 0.05), but not with viability or membrane integrity (p > 0.05). The correlation between motility and CPT2 gene expression was moderately positive, with a value of 0.5685 [48]. The expression of the CPT2 gene depends on the presence of double-chain unsaturated FAs, crucial for lipid metabolism. These acids are essential for both energy production and sperm motility regulation. Insufficient CPT2 gene expression hampers ATP synthesis, impairing sperm motility [55]. The CPT2 gene expression was not linked to membrane viability and integrity. The role of CPT2 gene expression in energy production is crucial and unaffected by membrane structure integrity or sperm cell viability [56]. The factors of cryopreservation, semen extenders, and thawing impact membrane integrity and sperm viability [5]. CPT2 gene expression varies among individuals, which is influenced by the availability of PUFA, which are the basic ingredients of lipid metabolism, as well as the integrity of DNA and mRNA, which play a role in the transcription and translation of CPT2 protein [55]. An extended thawing period of more than 10 s in 37°C water temperature can precipitate a surge in post-thaw ROS [57].

## Conclusion

This study demonstrated that adding CoQ10 to semen extenders decreases sperm damage and enhances sperm quality (viability, motility, membrane integrity, and ATP5F1A and CPT2 gene expression) after cryopreservation. In this study, we assessed sperm quality and gene expression in groups exposed to varying CoQ10 concentrations. This addition significantly influenced all examinations, suggesting a potential impact on multiple parameters. 25 mg/dL dose yielded the maximum viability examination value and ATP5F1A gene expression, exhibiting a moderate correlation. CPT2 expression shows the strongest correlation with motility, reaching its highest point at an additional 50 mg/dL dosage. The membrane integrity reached its highest values at additional doses of 25 and 50 mg/dL. The significance of CoQ10’s role in maintaining the expression of these genes requires further investigation.

## Authors’ Contributions

YO and IM: Conceptualization, methodology, and writing of the original draft. FARA and DDMP: Samples collection and laboratory assessment of sperm quality. VFH, SS, and TH: Analyzed data and performed statistical analyses. NYD: Data curation and finalization of the manuscript. All authors have read, reviewed, and approved the final manuscript.
